# Advances and Applications of *Clostridium* Co-culture Systems in Biotechnology

**DOI:** 10.3389/fmicb.2020.560223

**Published:** 2020-11-16

**Authors:** Yuanfen Du, Wei Zou, Kaizheng Zhang, Guangbin Ye, Jiangang Yang

**Affiliations:** ^1^College of Bioengineering, Sichuan University of Science and Engineering, Yibin, China; ^2^Research Laboratory of Baijiu Resource Microorgannisms and Big Data, Sichuan University of Science and Engineering, Yibin, China

**Keywords:** co-culture, *Clostridium*, hydrogen, butanol, interaction mechanism

## Abstract

*Clostridium* spp. are important microorganisms that can degrade complex biomasses such as lignocellulose, which is a widespread and renewable natural resource. Co-culturing *Clostridium* spp. and other microorganisms is considered to be a promising strategy for utilizing renewable feed stocks and has been widely used in biotechnology to produce bio-fuels and bio-solvents. In this review, we summarize recent progress on the *Clostridium* co-culture system, including system unique advantages, composition, products, and interaction mechanisms. In addition, biochemical regulation and genetic modifications used to improve the *Clostridium* co-culture system are also summarized. Finally, future prospects for *Clostridium* co-culture systems are discussed in light of recent progress, challenges, and trends.

## Introduction

Although axenic cultivation of individual-bacteria has been widely used in fermentation technology, many biological processes require the presence of multiple bacteria in a single system. For instance, co-culturing two or more microorganisms is an important and common fermentation technique that has been extensively applied to several bioprocesses, including food manufacturing (i.e., cheese, yogurt, sauerkraut, sourdough, kefir, salami, whisky, cacao beans, Belgian beer, etc.) (Bader et al., 2010), bio-degradation (i.e., wastewater treatment and biological soil remediation) (Yagi, 2011), and bio-fuel production (Luo et al., 2015). The greatest advantage of co-culture systems is that the combination of the metabolic capacity of two or more microorganisms allows for the utilization of more complex substrates and the production of specific products. In particular, cellulose from sources such as rice straw, wheat straw, corn cobs, corn stalk waste, and sweet sorghum stalk is not easily degraded and utilized. In addition, distillery effluent, crude glycerol, apple pomace, banana agro-waste, cassava starch, lactate, gelatin, yeast waste, various other carbohydrates, carbon monoxide and syngas can also be processed using these systems (Charubin et al., 2018). Inexpensive, renewable biomass and agricultural wastes from abundant renewable resources can be converted into bio-fuels and bio-solvents by pure bacterial cultures or by co-cultures of microorganisms. In co-culture systems, microorganisms stably coexist by interacting with each other and provide various functional factors or materials more effectively than single cultures, due to the synergistic involvement of the metabolic pathways of all involved strains (Wu et al., 2016).

Clostridia are Gram-positive, anaerobic or obligate anaerobic bacteria that are among a metabolically diverse group that includes cellulolytic, acetogenic, chain-elongating and solventogenic bacteria (Salimi et al., 2010; Charubin et al., 2018). Clostridia perform diverse metabolic functions, including the conversion of starch, protein, and purines into organic acids (i.e., acetic, butyric, and caproic acids), alcohols, CO_2_, and hydrogen (Zou et al., 2018b). Because of their broad and flexible metabolic capabilities, *Clostridium* species are included in many microbial co-culture ecosystems, which we will refer to in this review as “*Clostridium* co-culture systems.”

Typically, *Clostridium* co-culture systems are used to produce bio-fuels such as hydrogen and CH_4_, solvents, and organic acids (Jiang et al., 2019). Because cellulosic materials are commonly found in nature (Lu et al., 2017), the specific metabolic capacities of cellulolytic strains and producers in co-culture systems have attracted significant attention and offered many long-term prospects for development. In this review, we summarize recent progress on *Clostridium* co-culture systems, including their advantages, products, composition, interaction mechanisms, and regulation. Significant attention is paid to describing the regulation strategies based on an understanding of the interactions to improve the stability and fermentation performance of the *Clostridium* co-culture systems that have been used in large-scale bioprocesses and optimized for biotechnology applications.

## Advantages of *Clostridium* Co-Culture Systems

Compared to a mono-culture, there are many unique and essential advantages of the *Clostridium* co-culture systems. Firstly, co-culturing *Clostridium* spp. and other microorganisms makes it possible to utilize more extensive and complex substrates including the abundant renewable resources of lignocellulosic biomass in nature such as cedar (Rabemanolontsoa et al., 2016), aspen (Xu and Tschirner, 2014), agave (Oliva-Rodríguez et al., 2019), cassava (Qi et al., 2018), miscanthus biomass (Raut et al., 2019), switchgrass (Flythe et al., 2015), salix (Pang et al., 2018b), and many types of agricultural waste such as crop straw, fruit residue, etc. Food waste (Tavabe et al., 2010) and industrial waste including biodiesel waste (crude glycerol) and yeast waste can also be used.

Secondly, *Clostridium* co-culture systems can increase the efficiency of substrate utilization. For example, in the co-culture of *C. thermocellum* JN4 and *Thermoanaerobacterium thermosaccharolyticum* GD17, the cellulase complex of *C. thermocellum* JN4 can hydrolyze xylan to xylobiose and xylose but cannot utilize xylobiose or xylose, but *T. thermosaccharolyticum* GD17 can utilize these substrates to produce H_2_, organic acids and ethanol (Liu et al., 2008; Pang et al., 2018a, b). A co-culture system will also increase the substrate utilization rate when both bacteria can use the substrate to produce the target product (Pachapur et al., 2015a).

Thirdly, the *Clostridium* co-culture system can improve product yield. Hydrogen production by the co-culture of *Rhodobacter sphaeroides* and *C. butyricum* was enhanced by 160% compared with that by the mono-culture of *C. butyricum* (Zhu et al., 2001). In the co-culture of *C. beijerinckii* and *Geobacter metallireducens*, *G. metallireducens* utilized AQDS as an electron acceptor, regenerating AH_2_QDS, which caused changes in the intracellular NADH/NAD^+^ ratio and a metabolic shift from the butyric acid pathway to the acetic acid pathway. This enhanced the hydrogen molar yield, the hydrogen production rate, and the extent of xylose utilization by *C. beijerinckii* (Zhang et al., 2012).

The fourth advantage is the improvement of system robustness. On the one hand, co-cultures with facultative anaerobic or aerobic bacteria will consume oxygen during the co-culture and provide anaerobic conditions for *Clostridium* (Zuroff et al., 2013; Wushke et al., 2015; Mai et al., 2016; Ebrahimi et al., 2019; Oliva-Rodríguez et al., 2019). On the other hand, metabolites that inhibit *Clostridium* growth may be removed by co-culture partners. For example, formate, which inhibits the growth of *C. cellulolyticum*, could be consumed by the hydrogen-evolving bacterium *Citrobacter amalonaticus* and transformed into hydrogen (Zhang et al., 2016b). *Methanogen* 166 converted accumulated H_2_ that was produced by *C. kluyveri* H068 into methane, thereby eliminating the hydrogen-mediated feedback inhibition that normally constrains *C. kluyveri* H068 and thus enhancing caproic acid production (Yan and Dong, 2018).

A fifth advantage is that a *Clostridium* co-culture system can elongate the product synthesis chain. In addition to the use of complex organic carbohydrates, the *Clostridium* co-culture system can also use simple inorganic carbon, such as carbon monoxide and carbon dioxide, to synthesize polycarbonic organic acids or alcohols. For example, acetogenic Clostridia can convert CO, CO_2_ and syngas to ethanol and acetate that can be utilized by chain-elongating Clostridia or solventogenic Clostridia to produce medium-chain fatty acids (e.g., butyrate and caproate) and higher alcohols (e.g., butanol and hexanol and even octanol) in the acetogenic *Clostridium* co-culture (Diender et al., 2016; Richter et al., 2016; Youn, 2017; Li and Henson, 2019).

The sixth advantage is the scalability of *Clostridium* co-culture systems. In addition to laboratory-scale anaerobic bottle or shake bottle studies, larger fermentation tanks or bioreactors have been used in the *Clostridium* co-culture system and have been proved to have significant effects, like eliminating substrate inhibition (Pachapur et al., 2016a), increasing product yield (Zhang et al., 2012; Salimi and Mahadevan, 2013; Luo et al., 2017; Morsy, 2017; Kim et al., 2018; Cheng et al., 2019), showing good stability (Masset et al., 2012) and continuous and stable product generation (Barca et al., 2016; Wu et al., 2016).

In addition, a *Clostridium* co-culture system can reduce the cost of fermentation and simplify the process. On the one hand, the *Clostridium* co-culture system can utilize cheaper renewable substrates. On the other hand, co-culturing *Clostridium* with *Bacillus* or other facultative anaerobic or aerobic bacteria that are able to consume oxygen, providing an anaerobic environment for *Clostridium*, precludes the need for expensive reductants or anaerobic operating conditions or equipment (Mai et al., 2016; Srivastava et al., 2018; Ebrahimi et al., 2019; Oliva-Rodríguez et al., 2019). And growth needs can also be met without the addition of exogenous nutrients (Mori, 1990). In some cases, lignocellulose can be directly converted into products without the need for pretreatment (Islam et al., 2017; Sander et al., 2017). However, there are still some issues and challenges associated with the co-cultures, such as instability, toxic byproducts of the other species, greater efforts required for control, and a lack of established technology for large-scale industrial application.

## Microbial Composition and Products of *Clostridium* Co-Culture Systems

### Co-culture Systems of Cellulolytic Clostridia and Solventogenic Clostridia

Co-culturing cellulolytic *Clostridium* and solventogenic *Clostridium* species not only results in the degradation of complex lignocellulosic substrates, but can also produce high-value solvents and green hydrogen energy from cheap renewable resources (Table 1). In the co-culture system, the most extensively used cellulolytic Clostridia are *C. thermocellum*, *C. celevecrescens*, *C. cellulovorans*, and *C. termitidis*.

**TABLE 1:** Overview of *Clostridium* co-cultures of cellulolytic Clostridia and solventogenic Clostridia in biotechnology applications.

Applied micro-organisms	Inoculumratios/size	Substrate	Product/process	yield	References
*C. thermocellum* LQRI, *C. thermohydrosulfuricum*39E	1:1	Cellulosic Substrates	Ethanol	1.8 mol/mol a hydroglucose unit in cellulose	Ng et al., 1981
*C. thermocellum* YM4, *C. thermohydrosulfuricum* YM3	1:1	Avicel	Ethanol	1.96 mol/mol of a hydroglucose unit in cellobiose	Mori, 1990
*C. thermocellum*, *C. thermosaccharolyticum*, *C. thermohydrosulphuricurn*	The inoculum of all the strain were 5% (v/v) after the same culture time	Lignocellulosic substrates	Ethanol	2.9 g/L	Saddler and Chan, 1984
*C. thermocellum* ATCC27405, *C. thermolacticum* ATCC43739	1:1	Xylose	Ethanol	4.539 g/L	Xu and Tschirner, 2011
*C. thermocellum* CT2, *C. thermosaccharolyticum* HG8	All are 5% (v/v) inoculums after 24 h of culture	Banana agro-waste	Ethanol	22 g/L	Harish et al., 2010
*C. thermocellum* ATCC27405, *C. thermolacticum* ATCC43739	1:1	Cellobiose	Ethanol	4.8 g/L	Xu and Tschirner, 2014
*C. thermocellum* NRCC2688, *C. acetobutylicum* ATCC824	The inoculum of all the strain were 5% (v/v) after 3 days of culture	Lignocellulosic substrates	Butanol	0.3 g/L	Yu et al., 1985
*C. thermocellum* ATCC27405, *C. saccharoperbutylacetonicum* strain N1-4	1:1	Crystalline cellulose	Butanol	7.9 g/L	Nakayama et al., 2011
*C. cellulolyticum* ATCC35317, *C. acetobutylicum* ATCC824	1:1	Cellulose	Butanol	350 mg/L	Salimi and Mahadevan, 2013
*C. thermocellum* ATCC27405, *C. beijerinckii* NCIMB8052	Unavailable	Corncob	Butanol	8.75 g/L	Lin et al., 2013
*C. thermocellum* ATCC27405, *C. beijerinckii* NCIMB8052	The inoculums of strain ATCC27405 was 10% (v/v), and NCIMB8052 was 1% (v/v)	Alkali extracted corn cobs	ABE	ABE 19.9 g/L (acetone 3.96, butanol 10.9 and ethanol 5.04 g/L)	Wen et al., 2014b
*C. cellulovorans* 743B, *C. beijerinckii* NCIMB8052	10:1 (v/v)	Deshelled corn cobs	ABE	11.8 g/L solvents (2.64 g/L acetone, 8.30 g/L butanol and 0.87 g/L ethanol)	Wen et al., 2014a
*C. thermocellum* NBRC103400, *C. saccharoperbutylacetonicum* N1-4	1:1	Rice straw	Butanol	6.9 g/L	Kiyoshi et al., 2015
*C. celevecrescens* N3-2, *C. acetobutylicum* ATCC824	The inoculums of both strains were 2 mL	Cellulose	Butanol	3.73 g/L	Wang et al., 2015
*C. thermocellum* ATCC27405, *C. saccharoperbutylacetonicum* ATCC27021 N1-4	1:1	Switchgrass	Solvents	207 mg	Elia et al., 2016
*C. cellulovorans* ATCC35296, *C. beijerinckii* NCIMB8052	Unavailable	Mandarin orange wastes	Butanol	0.046 g/g dried strained lees	Tomita et al., 2019
*C. thermocellum* ATCC27405, *C. thermoaceticum* ATCC39073	1:1	Japanese cedar	Acetic acid	897 mg/L	Rabemanolontsoa et al., 2016
*C. thermocellum* ATCC27405, *C. thermoaceticum* ATCC39073	1:1	Glucose	Acetic acid	14.8 g/L	Rabemanolontsoa et al., 2017
*C. thermocellum* ATCC27405, *C. thermopalmarium* DSM5974	1:0.05	Cellulose	Hydrogen	1387 ml/L	Geng et al., 2010
*C. thermocellum* DSM7072, *C. thermosaccharolyticum* DSM869	1:0.25	Cornstalk waste	Hydrogen	68.2 mL/g cornstalk	Li and Liu, 2012
*C. thermocellum* DSM7072, *C. thermosaccharolyticum* DSM869	4:1	Cornstalk	Hydrogen	105.61 mL/g cornstalk	Li et al., 2014
*C. thermocellum* ATCC27405, *C. beijerinckii* ATCC51743	1:1	Switchgrass	Gas	680 mL gas	Flythe et al., 2015
*C. thermocellum* DSM7072, *C. thermosaccharolyticum* DSM572	1:1	Sweet sorghum stalk	Hydrogen	5.1 mmol/g-substrate	Islam et al., 2017
*C. termitidis* ATCC51846, *C. beijerinckii* DSM1820	1:1	Cellulose	Hydrogen	1.92 mol hydrogen/molhexose equivalent_added_	Gomez-Flores et al., 2017
*C. cellulovorans* ATCC35296, *C. acetobutylicum* CDBB-B1496	5:3	Lignocellulosic substrates	Hydrogen	128 mL/L	Valdez-Vazquez et al., 2019

In the early phase, Ng and his colleagues co-cultured *C. thermocellum* strain LQRI, which can hydrolyze alpha cellulose and hemicelluloses to produce ethanol and acetate, with *C. thermohydrosulfuricum* strain 39E, which can ferment glucose, cellobiose, and xylose to produce ethanol and acetate, and fermented a variety of cellulosic substrates. The ethanol yield observed was twofold higher than in *C. thermocellum* mono-culture fermentations (Ng et al., 1981). At the same time, a higher ethanol yield was obtained by a co-culture of *C. thermohydrosulfuricum* YM3 and *C. thermocellum* YM4, which was very stable and degraded avicel more rapidly than did strain YM4 mono-culture. Moreover, strain YM3 could replace yeast extract in supporting the growth of strain YM4 to produce a high ethanol yield (Mori, 1990). In addition, *C. thermosacchrolyticum* and *C. thermolacticum* were used in co-culture with *C. thermocellum* to produce ethanol (Saddler and Chan, 1984; Xu and Tschirner, 2011). Some agricultural wastes can also be directly converted to ethanol. In the co-culture system of *C. thermocellum* CT2 and *C. thermosaccharolyticum* HG8 fermented on alkali-treated banana-agro waste (leaves), *C. thermosaccharolyticum* utilized the xylose and pentose sugars formed during hemicellulose degradation by *C. thermocellum*, which are unable to be utilized by *C. thermocellum*. The co-culture showed higher ethanol production, improved cellulose degradation, and enhanced reducing sugars utilization and remained active even at substrate concentrations up to 100 g/L (obtaining a maximum ethanol yield of 22 g/L) (Harish et al., 2010).

*Clostridium thermocellum* can also be utilized to co-culture with *C. beijerinckii* or *C. acetobutylicum*. Solventogenic Clostridia use saccharides produced by *C. thermocellum* hydrolysis of lignocellulosic substrate for acetone-butanol-ethanol (ABE) fermentation (Yu et al., 1985; Lin et al., 2013; Wen et al., 2014b; Flythe et al., 2015). Wen and colleagues utilized alkali-extracted corn cobs as substrate and obtained 19.9 g/L ABE in the co-culture of *C. thermocellum* ATCC27405 and *C. beijerinckii* NCIMB8052 in a one-pot reaction (Wen et al., 2014b). In addition to the co-culture thermophilic cellulolytic Clostridia and solventogenic Clostridia for ABE fermentation, mesophilic cellulolytic Clostridia (e.g., *C. celevecrescens* N3-2, *C. cellulovorans* 743B) are also applied in co-culture for ABE fermentation (Wen et al., 2014a; Wang et al., 2015). Co-culture of *C. cellulovorans* 743B and *C. beijerinckii* NCIMB8052 degraded 68.6 g/L alkali-extracted, deshelled corn cobs and produced 11.8 g/L of solvents under optimized conditions (Wen et al., 2014a) and also demonstrated the ability of *C. beijerinckii* NCIMB8052 to utilize mandarin orange wastes in an isopropanol-butanol-ethanol fermentation (Tomita et al., 2019).

*Clostridium thermoaceticum* has also been co-cultured with *C. thermocellum* to produce a large amount of acetic acid from lignocellulose. Rabemanolontsoa and colleagues first reported the simultaneous conversion of cellulose-, hemicellulose-, lignin-derived compounds from biomass derived from hot-compressed water treatment of Japanese cedar into acetic acid. They employed a co-culture of *C. thermocellum* ATCC27405 and *C. thermoaceticum* ATCC39073, which can ferment the obtained products, together with other low-molecular-weight products such as monosaccharides, decomposed products, and organic acids, into acetic acid (Rabemanolontsoa et al., 2016). They found that non-sparged N_2_ and sparged CO_2_ promoted growth and acetic acid production of the co-culture *C. thermocellum* ATCC27405 and *C. thermoaceticum* ATCC39073, while sparged N_2_ inhibited both microorganisms, and produced 10.3 g/L of acetic acid from cellobiose under CO_2_ sparging.

In addition to co-culturing cellulolytic *Clostridium* and solventogenic *Clostridium* to produce solvents, hydrogen production has also been explored. *C. thermocellum* DSM1237, possessing the capacity to hydrolyze cellulose to produce hydrogen, was co-cultured with *C. thermopalmarium* DSM5974, which can produce hydrogen but cannot hydrolyze cellulose, and the co-culture produced nearly double the hydrogen production of *C. thermocellum* DSM1237 mono-cultures (Geng et al., 2010). At the same time, the co-culture of thermophilic *C. thermosaccharolyticum* DSM869 with *C. thermocellum* DSM7072, which can degrade cellulose and hemicellulose from cellulosic materials to produce soluble sugars, hydrogen, and acetic acid, also produced hydrogen from cornstalk waste (Li and Liu, 2012; Li et al., 2014). Further, *C. thermosaccharolyticum* DSM572, which can utilize soluble sugars to produce hydrogen, acetic acid, and butyric acid, co-cultured with *C. thermocellum* DSM7072 produced 5.1 mmol H_2_/g substrate, 1.27 g/L acetic acid, and 1.05 g/L butyric acid via thermophilic fermentation of sweet sorghum stalk (Islam et al., 2017). Moreover, mesophilic cellulolytic Clostridia have also been employed to produce hydrogen in a *Clostridium* co-culture system. The co-culture of *C. cellulovorans* ATCC35296 and *C. acetobutylicum* CDBB-B-1496 achieved a 2- to 3-fold improvement in hydrogen production in comparison with *C. acetobutylicum* CDBB-B-1496 mono-cultures from lignocellulosic substrates (Valdez-Vazquez et al., 2019). In addition, co-culturing *C. termitidis* ATCC51846, which can degrade cellulose to produce hydrogen, and *C. beijerinckii* DSM1820 achieved a high hydrogen yield of 1.92 mol/mol hexose equivalent_added_ compared to 1.45 mol/mol hexose equivalent_added_ in the mono-culture from cellulose (Gomez-Flores et al., 2017).

### Co-culture Systems of Celluloytic Clostridia With Other Microorganisms

Co-culturing cellulolytic Clostridia with other microorganisms has often been employed to produce hydrogen and ethanol (Table 2). *C. thermocellum*, *C. cellulovorans*, *C. cellulolyticum*, and *C. phytofermentans* were commonly used in co-culture. Different substrate utilizations were investigated in the co-culture system of *C. thermocellum* ATCC27405 and *Thermoanaerobacterium saccharolyticum* DSM571 to produce ethanol (Pang et al., 2018a, b). Among the co-cultures, *Thermoanaerobacterium saccharolyticum* DSM571 could utilize pentose and hexose, which were degraded and not used by *C. thermocellum* ATCC27405 to produce ethanol, but which could increase substrate utilization and product yield. In addition, the co-culture of *C. thermocellum* DSM1237 and *Caldibacillus debilis* GB1 achieved aerotolerant ethanogenic bio-fuel production of 5.5 mmol/L cell culture from cellulose (Wushke et al., 2015). At the same time, co-culturing the cellulolytic mesophile *C. phytofermentans* and *Candida molischiana* or *S. cerevisiae* cdt-1 achieved a more stable obligate mutualism for consortia-mediated lignocellulosic ethanol production by controlling the volumetric transport rate of oxygen. In the co-culture, both yeasts could provide respiratory protection to the obligate anaerobic *C. phytofermentans* and converted soluble carbohydrates released by cellulose hydrolysis to ethanol (Zuroff et al., 2013).

**TABLE 2:** Overview of *Clostridium* co-cultures of cellulolytic Clostridia in biotechnology applications.

Applied micro-organisms	Inoculum ratios/size	Substrate	Product/process	Yield/production rates	References
*C. thermocellum* ATCC35609, *Thermoanaerobacter pseudethanolicus* strain 39E	1:1	Cellulose	Ethanol	>60 mM	Qiang et al., 2011
*C. thermocellum*, *Thermoanaerobacterium saccharolyticum*	1:1	Avicel	Ethanol	38 g/L	Argyros et al., 2011
*C. phytofermentans*, *Saccharomyces cerevisiae* cdt-1	Unavailable	Cellulose	Ethanol	22 g/L	Zuroff et al., 2013
*C. thermocellum* DSM1237, *Caldibacillus debilis* GB1	1:1	Cellulose	Ethanol	4.2 mM	Wushke et al., 2015
*C. thermocellum* DSM1237, *Caldibacillus debilis* GB1	1:1	Cellulose	Ethanol	6.3 mmol/L	Wushke et al., 2015
*C. thermocellum* ATCC27405, *Thermoanaerobacterium thermosaccharolyticum* DSM571	1:5	Corn straw	Ethanol	0.45 g/L	Pang et al., 2018a
*C. thermocellum* ATCC27405, *Thermoanaerobacterium thermosaccharolyticum* DSM571	1:0.815	Salix	Ethanol	0.2 g/L	Pang et al., 2018b
*C. formicoaeetieum* ATCC27076, *Streptococcus lactis*	Unavailable	Whey lactose	Acetic acid	20 g/L	Tang et al., 1988
*C. thermocellum* JN4, *Thermoanaerobacterium thermosaccharolyticum* GD17	5:1	Cellulose	Hydrogen	1.8 mol/mol glucose	Liu et al., 2008
*C. cellulolyticum* H10, *R. palustris* CGA676	1:1	Cellulose	Hydrogen	1.4 mol/mol glucose	Jiao et al., 2012
*C. cellulovorans* 743B, *Rhodopseudomonaspalustris* CGA009	1:4	Cellulose	Hydrogen	12.2 ± 1.2 mL/day	Lu and Lee, 2015
*C. thermocellum* JN4, *T. thermosaccharolyticum* GD17	Unavailable	Cellulose	Hydrogen	0.024 mmol/h	Wang et al., 2016
*C. cellulolyticum* DSM5812, *Citrobacteramalonaticus*	1:1	Corn stover	Hydrogen	51.9 L H_2_/kg total solid	Zhang et al., 2016b
*C. thermoaceticum* 1745, *Methanosarcina sp.* CHTI55	1:1	Glucose	Methane	17.31 ml CH_4_/g cell/h	Nishio et al., 1990
*C. cellulovorans* ATCC35296, *M. barkeri* DSM804	1.4:1	Cellulose	Methane	0.87 ± 0.02 mol CH_4_/mol glucose equivalent	Lu et al., 2017
*C. cellulovorans* ATCC35296, *M. mazei* DSM3647	1.7:1	Cellulose	Methane	0.44 ± 0.04 mol CH_4_/mol glucose equivalent	Lu et al., 2017

In addition to producing ethanol via lignocellulose degradation, the co-culture of two thermophilic, anaerobic bacteria, *C. thermocellum* JN4 and *Thermoanaerobacterium thermosaccharolyticum* GD17, that were isolated from rotten wheat straw increased hydrogen production by about twofold compared to *C. thermocellum* JN4 mono-culture (Liu et al., 2008). In addition, mesophilic cellulolytic *C. cellulolyticum* DSM5812 was co-cultured with non-cellulolytic hydrogen-fermenting bacteria to produce hydrogen from corn stover. *Citrobacter amalonaticus* Y19 utilized glucose and xylose released by *C. cellulolyticum* hydrolysis of corn stover to produce hydrogen and consumed formate, which inhibits the growth of *C. cellulolyticum* and was transformed into hydrogen (Zhang et al., 2016b). Although cellulolytic Clostridia may play an economically relevant role in hydrogen production in anaerobic digesters from a high percentage of cellulosic materials, fermentative hydrogen yields are low on account of electrons lost during the obligate excretion of organic acids and alcohols. Jiao and colleagues artificially established a co-culture containing *C. cellulolyticum* H10 and the photosynthetic purple bacterium *Rhodopseudomonas palustris* CGA009 that can consume fermentation products such as acetic acid and butyric acid to produce H_2_ via nitrogenase during nitrogen fixation. In this co-culture, *C. cellulolyticum* could degrade all added cellulose (5.5 g/L) at a higher growth rate, and the total hydrogen production was 1.6 times higher than that produced by the *C. cellulolyticum* mono-culture (Jiao et al., 2012). And the co-culture system of *C. cellulovorans* 743B and *Rhodopseudomonas palustris* CGA009 obtained a H_2_ production rate of 12.2 ± 1.2 mL/day at optimized cellulose concentration (Lu and Lee, 2015).

The *Clostridium* co-culture system can also produce methane in addition to producing hydrogen and solvents, in particular the co-culture of cellulolytic Clostridia and methanogens including *Methanosarcina barkeri Fusaro*, *Methanosarcina mazei*, and *Methanothermobacter thermautotrophicus* (Sasaki et al., 2012; Lu et al., 2017). Lu et al. (2017) established two co-culture models combining *C. cellulovorans* 743B with *Methanosarcina barkeri Fusaro* or *Methanosarcina mazei*, that could directly convert cellulose into methane and obtained a CH_4_ yield of 0.87 ± 0.02 mol/mol glucose equivalent using cellulose in the co-cultures in which *C. cellulovorans* degraded cellulose to hydrogen, acetic acid, and formic acid, which could be converted by *M. barkeri* to produce methane.

### Co-culture Systems of Solventogenic Clostridia

#### Hydrogen Production

Solventogenic Clostridia are extensively used to produce hydrogen. Co-culture of two strains of solventogenic *Clostridium* was also studied (Table 3). Masset et al. used four pure Clostridia to compose three different co-cultures, (1) *C. felsineum* and *C. pasteurianum*, (2) *C. felsineum* and *C. butyricum*, (3) *C. pasteurianum* and *C. butyricum*, which produced hydrogen at higher rates than the mono-cultures, and the H_2_ yields were only slightly affected (Masset et al., 2012). In addition, Santilal explored the effect on hydrogen production by co-cultures of *C. beijerinckii* NCIMB6444 and *C. butyricum* NCIMB9578 from molasses and found that the accumulation of acetic acid and butyric acid severely inhibited the production of hydrogen. Hence, the co-culture was considered to create a possible negative interaction for hydrogen production (Santilal, 2015).

**TABLE 3:** Overview of *Clostridium* co-cultures of solventogenic Clostridia in biotechnology applications.

Applied micro-organisms	Inoculum ratios/size	Substrate	Product/process	Yield/production rates	References
*C. butyricum* IFO3847, *Rhodobacter sphaeroides* RV	1:1	Glucose	Hydrogen	Cumulative hydrogen 106 mL	Zhu et al., 2001
*C. butyricum* DSM10702, *R. sphaeroides* DSM158	1:5.9	Glucose	Hydrogen	0.60 mL hydrogen/mL medium	Fang et al., 2006
*C. pasteurianum* CH_4_, *Rhodopseudomonaspalustris* WP3-5	Unavailable	Sucrose	Hydrogen	14.2 mol/mol sucrose	Chen et al., 2008
*C. acetobutylicum* X_9_, *Ethanoigenensharbinense* B_49_	1:1	Microcrystalline cellulose	Hydrogen	1810 ml/L medium	Wang et al., 2008
*C. butyricum*, *Rhodopseudomonasfaecalis* RLD-53	1:5	Glucose	Hydrogen	4.134 mol/mol glucose	Ding et al., 2009
*C. acidisoli* sp. nov DSM12555, *R. sphaeroides* ZX-5	0.83	Sucrose	Hydrogen	10.16 mol/mol sucrose (5.08 mol/mol hexose)	Sun et al., 2010
*C. acetobutylicum*, *R. sphaeroides*	1:2	Food waste	Hydrogen	22.7 L	Tavabe et al., 2010
*C. butyricum* TISTR1032, *Enterobacter. aerogenes* TISTR1468	1:1	Cassava pulp	Hydrogen	3385 ml H_2_/L day and 345.8 ml H_2_/g COD_reduced_	Phowan et al., 2010
*C. beijerinckii* L9, *C. butyricum* M1 and *B. thermoamylovorans* B5	8.9:4.8:10.3	Yeast waste	Hydrogen	46 mL H_2_/g COD added yeast waste	Chou et al., 2011
*C. butyricum*, *E. coli* K-12 MG1655	Unavailable	Glucose	Hydrogen	1.65 mol/mol glucose	Seppälä et al., 2011
*C. felsineum* DSM749, *C. pasteurianum* DSM525	1:1	Glucose	Hydrogen	1.5 L biogas/h	Masset et al., 2012
*C. butyricum*, *R. sphaeroides*	Unavailable	Glucose	Hydrogen	15.9 mL H_2_/L/h	Lee et al., 2012
*C. beijerinckii* NCIMB8052, *Geobacter metallireducens* GS-15	3:1	Xylose	Hydrogen	198.2 ± 10.9 μmol	Zhang et al., 2012
*C. beijerinckii* NCIMB8052, *Geobacter metallireducens*	Unavailable	Xylose	Hydrogen	287 ± 9 μmol	Zhang, 2013; Zhang et al., 2013
*C. butyricum* ATCC19398, *R. palustris* ATCC17001	1:10	Sucrose	Hydrogen	Cumulative hydrogen 830 mL	Kao et al., 2014
*C. beijerinckii* NCIMB6444, *C. butyricum* NCIMB9578	1:1	Molasses	Hydrogen	23.48 mL/L/h	Santilal, 2015
*C. butyricum*, *R. sphaeroides* N7	1:1	Glucose	Hydrogen	4.9 mol/mol hexose	Laurinavichene and Tsygankov, 2015
*C. acetobutylicum* DSM792, *R. sphaeroides* ATCC49419	1:2	Glucose	Hydrogen	6.22 mol/molglucose	Zagrodnik and Laniecki, 2015
*C. acetobutylicum* X9, *Ethanoigenens harbinense* B2	1:1	Microcrystalline cellulose	Hydrogen	10.4 mmol/g MCC	Bao et al., 2016
*C. butyricum* NRRLB-41122, *E. aerogenes* NRRLB-407	1:1	Crude glycerol	Hydrogen, ethanol and 1,3-propanedio	26.14 mmol H_2_/L, 1.4 g ethanol/L, 0.5 g 1,3-propanediol/L	Pachapur et al., 2015a
*C. butyricum* NRRLB-41122, *E. aerogenes* NRRLB-407	15%	Crude glycerol and apple pomace hydrolyzate	Hydrogen	26.07 ± 1.57 mmol/L	Pachapur et al., 2015b
*C. butyricum* NRRLB-41122, *Enterobacter aerogenes* NRRLB-407	1:1	Crude glycerol and eggshell biowaste	Hydrogen	31.66 ± 0.55 mmol/L	Pachapur et al., 2016a
*C. butyricum* NRRLB-41122, *Enterobacter aerogenes* NRRLB-407	1:1	Biodiesel waste	Hydrogen	32.1 ± 0.03 mmol/L	Pachapur et al., 2016b
*C. butyricum* ATCC19398, *Rhodopseudomonas palustris* ATCC17001	1:1	Sucrose	Hydrogen	2.16 mol/mol sucrose	Kao et al., 2016a
*C. butyricum* ATCC19398, *Rhodopseudomonas palustris* ATCC17001	1:1	Sucrose	Hydrogen	5.42 mol/mol sucrose	Kao et al., 2016b
*C. butyricum* N1VKMB-3060, *R. sphaeroides* VKMB-3059	Unavailable	Starch	Hydrogen	6.2 mol/mol glucose	Laurinavichene et al., 2016
*C. butyricum*, *R. sphaeroides* N7	Unavailable	Starch	Hydrogen	6.1 mol/mol glucose	Laurinavichene and Tsygankov, 2016
*C. butyricum* MIYAIRI, *R. sphaeroides* RV	3:10	Glucose	Hydrogen	8.71 mL/h	Takagi et al., 2016
*C. acetobutylicum*, *Desulfovibrio vulgaris*	1:1	Glucose	Hydrogen	1.20–1.34 mol/mol glucose	Barca et al., 2016
*C. beijerinckii* DSM1820, *C. saccharoperbutylacetonicum* DSM14923	1:1	Glucose	Hydrogen	2.69 mol/mol glucose	Nasr et al., 2017
*C. butyricum* NRRL-B 1024, *R. palustris* GCA009	1:3	Potato starch/glucose	Hydrogen	6.4 ± 1.3 mol/mol glucose	Hitit et al., 2017a
*C. butyricum* NRRL-B-1024, *E. aerogenes* NRRL-B-115, *R. palustris* GCA009	Unavailable	Starch/Glucose	Hydrogen	8.3 ± 0.1 mmol H_2_/g COD	Hitit et al., 2017b
*C. acetobutylicum* DSM792, *R. sphaeroides* ATCC49419	1:2	Corn starch	Hydrogen	2.62 mol/mol hexose	Zagrodnik and Łaniecki, 2017b
*C. acetobutylicum* DSM792, *R. sphaeroides* ATCC49419	1:2	Starch	Hydrogen	5.11 mol/mol glucose	Zagrodnik and Łaniecki, 2017a
*C. acetobutylicum* ATCC824, *Rhodobacter capsulatus* DSM1710	Unavailable	Molasses	Hydrogen	5.65 mol/mol hexose	Morsy, 2017
*C. butyricum* NRRLB-41122, *Enterobacter aerogenes* NRRLB-407	2:1	Biodiesel industry waste	Hydrogen	19.46 ± 0.95 mmol/L	Pachapur et al., 2017
*C. butyricum* N1VKMB-3060, *R. sphaeroides* VKMB-3059	1:2	Starch	Hydrogen	5.2 mol/mol glucose	Laurinavichene et al., 2018
*C. sporogenes* ATCC19404, *E. aerogenes* ATCC13048	Unavailable	Pineapple Biomass Residue	Hydrogen	35.9 mmol/h/L substrate	Jalil et al., 2018
*C. pasteurianum* MTCC116, *Bacillus subtilis* PF_1	1:1	Sugarcane bagasse hydrolyzate	Hydrogen	2870 mL/L	Srivastava et al., 2018
*C. beijerinckii* ST1, *C. bifermentans* ST4, and *C. butyricum* ST5	1:1:1	Sucrose	Hydrogen	1.13 ± 0.015 L/L medium	Hoang et al., 2018
*C. butylicum* TISTR1032, *B. subtilis* WD161	1:1	Cassava starch	ABE	9.71 g/L	Tran et al., 2010
*C. butylicum* TISTR1032, *B. subtilis* WD161	Unavailable	Cassava starch	ABE	9.02 ± 0.17 g/L	Tran et al., 2011
*C. acetobutylicum* ATCC824, *S. cerevisiae*	Unavailable	Glucose	Butanol	15.74 g/L	Luo et al., 2015
*C. acetobutylicum* ATCC824, *S. cerevisiae*	Unavailable	Glucose	Acetone	8.55 g/L	Luo et al., 2015
*C. acetobutylicum* TSH1, *B. cereus* TSH2	10:1	Glucose	ABE	18.1 g/L	Wu et al., 2016
*C. beijerinckii* ATCC6014, *C. tyrobutyricum* ATCC25755	Unavailable	Cassava bagasse	Isopropanol and *n*-butanol	The yield of isopropanol and butanol were 7.63 and 13.26 g/L, respectively	Zhang et al., 2016a
*C. acetobutylicum* ATCC824, *S. cerevisiae*	Unavailable	Corn flour	N-butanol	16.3 g/L	Luo et al., 2017
*C. beijerinckii* NCIMB8052, *Bacillus cereus* CGMCC1.895	1:1	Corn mash	Butanol	10.49 g/L	Mai et al., 2016
*C. acetobutylicum* CH02, *S. cerevisiae*	1:1	Cassava	Solvents	0.46 g solvents/g glucose	Qi et al., 2018
*C. acetobutylicum* TSH1, *B. cereus* TSH2	1:1	Glucose	Butanol	12.3 ± 0.9 g/L	Mi et al., 2018
*C. beijerinckii* F-6, *S. cerevisiae*	1:1	Glucose	Butanol	12.75 g/L	Wu et al., 2019
*C. acetobutylicum* ATCC824, *Bacillus subtilis* CDBB555	2:3	Agave hydrolyzates	Butanol	8.28 g/L	Oliva-Rodríguez et al., 2019
*C. acetobutylicum* PTCC1492, *Nesterenkonia* sp. strain F	15:1	Glucose	Butanol	13.6 g/L	Ebrahimi et al., 2019
*C. acetobutylicum* ATCC824, *Fibrobacter succinogenes* S85	3:2	Miscanthus biomass	Solvents	0.091 g/g	Raut et al., 2019
*C. acetobutylicum* ATCC824, *C. tetanomorphum* ATCC49273	6.5:6.5	Sweet sorghum juice	Lactic acid	2.7 g/L	Ndaba et al., 2015
*C. tyrobutyricum* ATCC25755, *M. hexanoica*	1:1	Fructose	Caproic acid	0.69 g/L/h	Kim et al., 2018

Combining heterotrophic anaerobic bacteria and anoxygenic phototrophic bacteria to produce hydrogen has attracted great attention, especially the co-culture of Clostridia with strains of *Rhodopseudomonas* or *Rhodobacter*. *Clostridium* spp. can use complex carbohydrates to produce organic acids and hydrogen through anaerobic digestion. In return, these organic acids can be utilized to increase hydrogen production by *Rhodopseudomonas* or *Rhodobacter*. Co-culture of *Clostridium butyricum* IFO3847 and *Rhodobacter sphaeroides* RV enhanced hydrogen production by 160% compared to the mono-culture of *C. butyricum*. However, research found that NH_4_^+^ affected the accumulation of the reducing power that is essential for hydrogen production, and thus affected the hydrogen yield (Zhu et al., 2001). Chen et al. (2008) used a two-stage fermentation process in which the soluble metabolites produced by dark H_2_ fermentation were appropriately prediluted to avoid NH_4_^+^ concentration inhibition before further use by photo fermentation in the co-culture of *C. pasteurianum* CH_4_ and *Rhodopseudomonas palustris* WP3-5. They achieved a stable continuous culture with an average H_2_ yield of 10.21 mol/mol sucrose for nearly 10 days. Furthermore, the effect of the total volatile fatty acids concentration was greater than that of the NH_4_-N concentration (Kao et al., 2016a). At the same time, Laurinavichene and Tsygankov (2015) observed the inhibition of *Clostridium* by purple bacteria in which the purple bacteria could consume hydrogen produced by *C. butyricum* at early phase, thus reducing the hydrogen yield. Further, they found hydrogen production by *C. butyricum* in co-culture with *Rhodobacter sphaeroides* N7 was retarded as compared to the mono-culture after 3 days (Laurinavichene and Tsygankov, 2015) and confirmed that a high yeast extract concentration (160–320 mg/L) enhanced reliable H_2_ production (Laurinavichene and Tsygankov, 2016; Laurinavichene et al., 2016). They used a repeated batch photofermentation utilizing starch with co-culture of *C. butyricum* and *R. sphaeroides* N7 over 15 months to obtain efficient H_2_ production with an average H_2_ yield of 5.2 mol/mol glucose with no accumulation of organic acids in the presence of Clostridia (Laurinavichene et al., 2018). The highest hydrogen yield of 6.4 ± 1.3 mol/mol glucose was achieved upon optimization of the culture conditions using response surface methodology in the co-culture system of *C. butyricum* NRRLB1024 and *Rhodospeudomonas palustris* GCA009 (Hitit et al., 2017a). Co-culturing *C. acidisoli* sp. nov DSM12555 and *R. sphaeroides* ZX-5 also gaved a relatively high hydrogen yield (Sun et al., 2010). In addition, *C. acetobutylicum* was extensively utilized in co-culture with *Rhodobacter* or *Rhodopseudomonas* species to produce hydrogen. The co-culture of *C. acetobutylicum* DSM792 and *R. sphaeroides* ATCC49419 produced a high H_2_ yield of 6.22 mol/mol glucose utilizing glucose under optimal conditions (Zagrodnik and Laniecki, 2015).

In addition to the anaerobic photosynthetic bacteria, which remove metabolic inhibitors of Clostridia and increase hydrogen production, other dark-fermentative hydrogen-producing bacteria such as *Ethanoligenens harbinense* (Wang et al., 2008), *Escherichia coli* (Seppälä et al., 2011), *Bacillus*, and *Enterobacte*r were also considered as partners to co-culture with Clostridia. Not only can each of these bacteria be used as a biological fortifier to promote the production of hydrogen, but they also use a wide range of substrates, which has attracted wide attention. *C. acetobutylicum* X_9_, which can degrade microcrystalline cellulose with a typical butyrate-type fermentation metabolism and acetate and butyrate as primary metabolites, was co-cultured with *Ethanoigenens harbinense* B_49_, which can produce hydrogen efficiently utilizing reduced sugar from the cellulose hydrolysis of X_9_. The co-culture hydrolyzed more cellulose and increased hydrogen production rates compared with the mono-culture of X_9_ using microcrystalline cellulose (Wang et al., 2008). Seppälä et al. (2011) co-cultured *C. butyricum* and *Escherichia coli* K-12 MG1655, which more efficiently utilized glucose although hydrogen production was lower than for *C. butyricum* mono-culture. In addition, facultative anaerobic *Enterobacte*r aerogenes, which can act as a reducing agent to consume oxygen creating an anaerobic environment for obligate anaerobic Clostridia, has been extensively applied in a *Clostridium* co-culture system. Phowan et al. (2010) used the co-culture of *C. butyricum* TISTR1032 and *E. aerogenes* TISTR1468 and found that it could reduce the lag time and produce hydrogen from cassava pulp hydrolyzate without any reducing agents in the medium. Moreover, co-culture of *Enterobacter aerogenes* and *C. butyricum* could also use crude glycerol and obtained a hydrogen production of 26.07 ± 1.57 mmol H_2_/L of medium (Pachapur et al., 2015b). In addition, facultative anaerobic *Bacillus* was also utilized in a *Clostridium* co-culture. *B. thermoamylovorans* was found not only to consume oxygen to produce anaerobic conditions, but it also hydrolyzed complex substrates by secreting a variety of extracellular enzymes such as cellulase, α-amylase, lipase, protease, and pectinase to stimulate the production of hydrogen by *C. beijerinckii* L9 from yeast waste (Chang et al., 2008; Chou et al., 2011).

In addition to co-culture with photosynthetic bacteria and facultative anaerobes to produce hydrogen, co-culture with *Geobacter metallireducens* also demonstrated significant hydrogen production. Zhang found that co-culturing *G. metallireducens* GS-15 and *C. beijerinckii* NCIMB8052 increased hydrogen production by 52.3%, and the extent of substrate utilization by 39.0% (Zhang et al., 2012). In addition, Barca et al. (2016) achieved a stable H_2_ production of 1.23 ± 0.23 mol/mol glucose after 3–4 days of operation using an up-flow anaerobic biofilm reactor by the co-culture of *C. acetobutylicum* ATCC824 and *Desulfovibrio vulgaris*. *C. bifermentans* ST4, newly isolated from cow rumen, was co-cultured with *C. beijerinckii* ST1 and *C. butyricum* ST5 to obtain hydrogen production of 1.13 ± 0.015 L/L medium (Hoang et al., 2018). These studies suggest that the development of new co-culture systems for Clostridia offers great potential for improving hydrogen production.

#### Solvent Production

*Clostridium acetobutylicum*, *C. butyricum*, and *C. beijerinckii* are typical solventogenic Clostridia that engage in ABE fermentation (Table 3). In a co-culture of solventogenic Clostridia and *Bacillus*, *Bacillus* not only provides an anaerobic environment for Clostridia by removing oxygen, but also increases the hydrolysis of complex substrates to promote the production of solvents, especially butanol (Tran et al., 2010; Tran et al., 2011; Wu et al., 2016; Mai et al., 2016; Oliva-Rodríguez et al., 2019). The co-culture of *C. acetobutylicum* TSH1 and *Bacillus cereus* TSH2 produced 11.2 g/L butanol under microaerobic conditions (Wu et al., 2016). Higher butanol yield was obtained by co-culturing solventogenic Clostridia and *Saccharomyces cerevisiae*. *S. cerevisiae* can not only use fermentable sugars to produce ethanol, but also secrete amino acids assimilated in and utilized by Clostridia to enhance the tolerance of Clostridia to higher butanol concentrations and increase of solvent yield (Luo et al., 2015, 2016, 2017; Qi et al., 2018; Wu et al., 2019). Luo et al. achieved high total ABE concentrations of 24.8 g/L including 16.3 g/L of butanol via the added butyrate fermentative supernatant produced by *C. tyrobutyricum*, which can produce butyric acid from fermentable sugars in the *C. acetobutylicum* ATCC824 and *Saccharomyces cerevisiae* co-culture (Luo et al., 2017). Moreover, *C. tyrobutyricum* was also co-cultured with *C. beijerinckii* ATCC6014 to increase butanol production (Zhang et al., 2016a). Recently, Ebrahimi et al. (2019) found the co-culture of *C. acetobutylicum* and *Nesterenkonia* sp. strain F gave an efficient yield of 13.6 g/L butanol under aerobic conditions, which further made it possible to avoid control of anaerobic conditions.

Notably, biological saccharification proved to be as promising as thermodynamic saccharification, which is of great significance for the direct production of target products using lignocellulosic materials. Raut utilized *Fibrobacter succinogenes* S85 with highly efficient saccharification ability found in the herbivore rumen, co-cultured with *C. acetobutylicum* ATCC824, and achieved a yield of 0.091 g/g total solvents from miscanthus biomass. This provides a promising approach using biological saccharification to make lignocellulosic bio-fuels (Raut et al., 2019). In addition, co-culture of solventogenic Clostridia and other bacteria can also produce lactic acid (Ndaba et al., 2015), butyric acid, and caproic acid. The co-culture of *C. tyrobutyricum* and *Bacillus sp.* SGP1 gave a high yield of butyric acid (34.2 ± 1.8 g/L) from sucrose (Dwidar et al., 2013). Moreover, *C. tyrobutyricum* can also be used to produce caproic acid. For example, the co-culture of *C. tyrobutyricum* and *Megasphaera hexanoica*, which is capable of producing caproic acid from acetic and butyric acids, gave10.08 g/L caproic acid using submerged hollow-fiber membrane bioreactors (Kim et al., 2018).

### Co-culture Systems of Acetogenic Clostridia and Chain-Elongating Clostridia

Acetogenic Clostridia such as *C. autoethanogenum* and *C. ljungdahli* can convert CO_2_ or syngas to ethanol and acetate by employing the Wood-Ljungdahl pathway (WLP), which is the most effective and remarkable non-photosynthetic carbon fixation pathway. In the WLP, two molecules of CO_2_ are reduced (by H_2_) to form one molecule of acetyl-CoA (Abrini et al., 1994; Charubin et al., 2018). *C. kluyveri* is a typical chain-elongating *Clostridium* that employs reversed β-oxidation reactions to convert short chain fatty acids and ethanol into medium chain fatty acids and hydrogen (Charubin et al., 2018; Zou et al., 2018a). Co-culturing an acetogenic *Clostridium* and a chain-elongating *Clostridium* enables direct conversion of CO_2_ or syngas into high-value medium chain fatty acids (Table 4). Diender et al. established a co-culture of *C. autoethanogenum* DSM10061 and *C. kluyveri* DSM555 using solely CO or syngas as the substrate and obtained butyrate and caproate, and butanol and hexane. Furthermore, they found that caproate toxicity is one of the severe culture problems and that controlling the pH or supporting a continuous process may improve production (Diender et al., 2016).

**TABLE 4:** Overview of *Clostridium* co-cultures of acetogenic Clostridia or chain elongating Clostridia in biotechnology applications.

Applied micro-organisms	Inoculum ratios/size	Substrate	Product/process	Yield/production rates	References
*C. autoethanogenum* DSM10061, *C. kluyveri* DSM555	1:1	Carbon monoxide or syngas	Medium-chain fatty acids and higher alcohols	Butyrate and caproate were at a rate of 8.5 ± 1.1 and 2.5 ± 0.63 mmol/L/day, butanol and hexanol at a rate of 3.5 ± 0.69 and 2.0 ± 0.46 mmol/L/day	Diender et al., 2016
*C. ljungdahlii* PETC, *C. kluyveri* DSM555	2:1	Syngas	Butanol, hexanol, and octanol	The net volumetric production rates of *n*-butanol, *n*-hexanol, and *n*-octanol were 39.2, 31.7, and 0.045 mmol/L/d, respectively	Richter et al., 2016
*C. carboxidivorans* NCIMB15243, *C. beijerinckii* NCIMB8052	1:1	CO_2_ and H_2_	Butanol	2.309 g/L	Youn, 2017
*C. aceticum*, *C. cellulovorans*	Unavailable	Cellulose	Acetate	1.011 g/L	Xia et al., 2017
*C. kluyveri* H068, *Methanogen* 166	2:1	Acetate and ethanol	Caproic acid	435.72 ± 13.58 mg/100 mL	Yan and Dong, 2018
*C. autoethanogenum* DSM10061, *C. kluyveri* DSM555	1:20	Syngas	Butyrate and caproate	The butyrate and caproate concentrations were 5.5 ± 0.7 mM and 1.3 ± 0.3 mM, respectively	Diender et al., 2019

Furthermore, co-culturing acetogenic Clostridia or chain-elongating Clostridia with other microorganisms has proved to have great potential in improving solvent production performance. In the co-culture of *C. kluyveri* H068 and *Methanogen* 166 from ethanol and acetate, the *Methanogen* 166 strain converted accumulated hydrogen produced by *C. kluyveri* H068 into CH_4_, thus eliminating the hydrogen-mediated feedback inhibition of *C. kluyveri* H068 and enhancing caproic acid production (Yan and Dong, 2018). *C. aceticum* is a homoacetogen that grows chemolithotrophically from H_2_ and CO_2_ and achieves product yield stoichiometrically via the WLP, and it was co-cultured with cellulolytic *C. cellulovorans*. This system utilized cellulosic biomass to produce solvents and an acetate yield of 1.011 g/L was obtained upon optimization of the pH (Xia et al., 2017). In addition, co-culturing with solventogenic Clostridia has also been investigated. Co-culturing *C. carboxidivorans* NCIMB15243, which can utilize CO_2_ and H_2_ via the reductive acetyl-CoA pathway or the WLP to produce organic acids, such as acetate and butyrate, and alcohols, such as ethanol and butanol, and *C. beijerinckii* NCIMB8052 achieved ethanol and butanol production from CO_2_ and H_2_ (Youn, 2017). Li and Hense (2019) co-cultured *C. autoethanogenum* and the human gut bacterium *Eubacterium rectale*, which can convert acetic acid into butyric acid, improving the butyrate productivity from carbon monoxide.

## Microbial Interaction Mechanisms in *Clostridium* Co-Culture Systems

### Methods Used to Study Microbial Interactions

Monitoring the biomass of bacteria in a *Clostridium* co-culture system is considered to be an effective strategy for monitoring the stability of the system. In addition to the traditional plate culture method of counting colony-forming units (CFU), which is time consuming, and fluorescence microscopy counting (Jiao et al., 2012), which is unable to distinguish bacteria that share similar morphology, the fluorescence *in situ* hybridization (FISH) method (Fang et al., 2006), spectrophotometer detection (Ding et al., 2009; Xu and Tschirner, 2014), qPCR analysis method (Geng et al., 2010; Sun et al., 2010; Zhang et al., 2012; Salimi and Mahadevan, 2013; Wen et al., 2014a, b; Benomar et al., 2015; Lu and Lee, 2015; Wu et al., 2016) have been used to analyze the dynamic abundances of microorganisms in a *Clostridium* co-culture system.

In addition, other molecular techniques have been used for systematic monitoring to elucidate interactions or to obtain metabolic strategies for improving the fermentation performance of the *Clostridium* co-culture system. Luo et al. utilized a real-time fluorescence quantitative PCR analysis method to measure the transcriptional levels of the *ctfAB* gene in different batches of *C. acetobutylicum* in a co-culture. They found that the transcriptional levels of *ctfAB* significantly increased with exogenous acetate addition as compared with those of the control, which showed that the increases of *ctfAB* transcriptional levels also enhanced acetone bio-synthesis in ABE fermentations (Luo et al., 2015). Lu et al. (2016) used next-generation sequencing technology to explore the global transcriptomic responses of the co-culture obtaining *C. cellulovorans* 743B and *Rhodopseudomonas palustris* CGA009. They analyzed and compared gene expression levels of in the co-cultures and the mono-cultures during the early-, mid-, and late-exponential growth phases respectively via genome-wide transcriptome sequencing. The results indicated the nitrogen fixation genes of *R. palustris* CGA009 and the cellulosomal genes of *C. cellulovorans* 743B were upregulated in co-culture, which contributed to a better understanding and optimization of the hydrogen-producing co-culture systems (Lu et al., 2016). In addition, RT-PCR was used to analyze the expression levels of key pathway genes to better understand the interactions in the *Clostridium* co-culture system (Benomar et al., 2015; Wang et al., 2016; Lu et al., 2017). Furthermore, flow cytometry, fluorescence microscopy, scanning electron microscopy (SEM), and a specific genetic marker were also utilized to elucidate bacterial strain interactions in the *Clostridium* co-culture system (Benomar et al., 2015; Pachapur et al., 2016a). Charubin and Papoutsakis established a transwell system utilizing a permeable membrane allowing metabolite exchange between both compartments and used quantitative PCR (qPCR) for population analysis and RT-qPCR for gene expression analysis. They found that unique, direct cell-to-cell interactions between *C. acetobutylicum* and *C. ljungdahlii* led to electron exchange and metabolite exchange, and changed the gene-expression patterns between partner organisms (Charubin and Papoutsakis, 2019). Sander et al. (2017) developed and validated an effective protocol to determine selected redox- and energy-related metabolite concentrations, including intracellular NAD(H), NADP(H), ATP, ADP, and AMP in *C. thermocellum* and *T. saccharolyticum* co-culture, which can clarify the redox state of these cofactors and help identify a strategy to achieve higher bioproductivity. Moreover, proteomic analysis and comparative transcriptome analysis were also applied to the *Clostridium* co-culture system to obtain strategies for improving fermentation performance (Mi et al., 2018; Diender et al., 2019).

### Use of Complementary Metabolic Pathways

**FIGURE 1:**
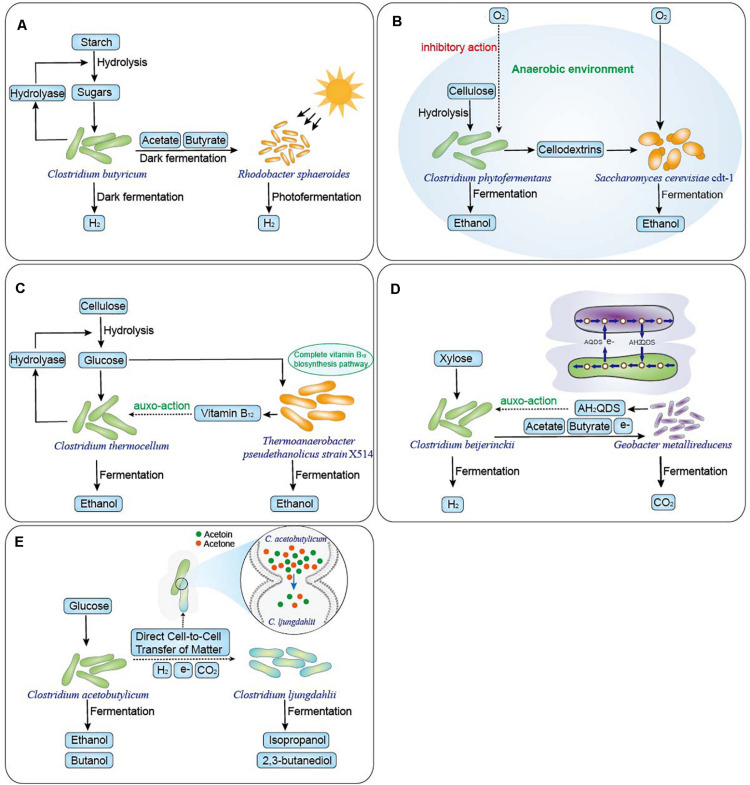
Schematic diagram of five type microbial interaction mechanisms which were shown to improve *Clostridium* co-culture systems. **(A)** Use of complementary metabolic pathways. *Rhodobacter sphaeroides* converts the acetate and butyrate produced by *C. butyricum* into hydrogen (6.2 mol H_2_/mol glucose) by photo fermentation from starch (Laurinavichene et al., 2016). **(B)** Removal of metabolic inhibitors. Yeast consume oxygen, creating an anaerobic environment in which *C. phytofermentans* produces ethanol from cellulose and obtaining high ethanol yield of 22 g/L with stable symbiotic relationship by controlling oxygen transport of approximately 8 μmol/L hour (Zuroff et al., 2013). **(C)** Cofactor complementation. *Thermoanaerobacter* strains X514 with a complete vitamin B_12_ biosynthesis pathway significantly enhances ethanolic fermentation of cellulolytic *Clostridium thermocellum* (Qiang et al., 2011). **(D)** Interspecies hydrogen and electron transfer. *G. metallireducens* oxidizes acetate and butyrate to regenerate AH_2_QDS, significantly improving *C. beijerinckii* fermentation of hydrogen from xylose (Zhang et al., 2012). **(E)** Direct cell-to-cell material exchange. Direct cell-to-cell interactions and material exchange of acetone and acetoin between *C. acetobutylicum* ATCC824 and *C. ljungdahlii* ATCC55383 with direct electron transfer results in non-native metabolites production (Charubin and Papoutsakis, 2019).

Most of the systems discussed entail a synergistic interaction in the *Clostridium* co-culture system. Understanding microbial interactions will lead to improvements in the monitoring and control over the system stability. It is the complementary metabolic pathways that exist in many co-culture systems that achieve the conversion of substrates into target products. In this case, one bacterium may convert complex macromolecular substrates such as lignocellulosic raw materials, starchy raw materials, crude glycerol, or gases such as carbon monoxide and syngas into substrates such as soluble sugars and acids, which also function as nutrients or precursors that can be used by the companion bacterium to produce the desired final products. For example, cellulolytic Clostridia degrade complex lignocellulosic substrates into soluble sugars that can be used by solventogenic Clostridia to produce solvents. Anoxygenic phototrophic bacteria such as *Rhodopseudomonas* or *Rhodobacter* utilize acetic acid and butyric acid produced by solventogenic Clostridia from soluble saccharides such as glucose, sucrose, and starch to produce hydrogen (Figure 1A). Or in the co-culture system of an acetogenic *Clostridium* and a chain-elongating *Clostridium*, the acetogenic *Clostridium* converts CO_2_ or syngas to ethanol and acetate employing the WLP, and the chain-elongating *Clostridium* such as *C. kluyveri* converts short-chain fatty acids plus ethanol into medium-chain fatty acids, such as butyrate and caproate, and higher alcohols and hydrogen via a reversed β-oxidation metabolism. Complementary interactions of metabolic pathways broaden the scope of substrate utilization and can produce target products more economically.

### Removal of Metabolic Inhibitors

As Clostridia are strict anaerobic bacteria, the removal of oxygen by associated bacteria suppresses oxygen toxicity and plays a necessary role in co-culture and stable fermentation. This type of microbial co-culture system provides protection from environmental influences (Bader et al., 2010). It has been reported that many types of associated bacteria such as *Bacillus*, *Enterobacter*, *Saccharomyces cerevisiae* (Figure 1B) (Zuroff et al., 2013), *Candida molischiana* (Zuroff et al., 2013), *Caldibacillus debilis* (Wushke et al., 2015), and *Nesterenkonia sp. F* (Ebrahimi et al., 2019) have the ability to remove oxygen, creating anaerobic conditions for *Clostridium*. In addition to removing oxygen to maintain an anaerobic environment, the removal of other metabolic inhibitors plays a critical role in the stability of a co-culture. For instance, in co-culture systems of *C. cellulolyticum* and *C. amalonaticus*, *C. cellulolyticum* can hydrolyze corn stover to release glucose and xylose, which are quickly utilized by *C. amalonaticus* to produce hydrogen. Formate, an inhibitor of *C. cellulolyticum* growth, is also consumed by *C. amalonaticus* and transformed into hydrogen (Zhang et al., 2016b).

### Cofactor Complementation

Cofactors are non-protein compounds that combine with enzymes and reactants to promote their activation and accelerate enzymatic reactions (Qin et al., 2009). Sato et al. (1992) confirmed that Vitamin B_12_ is beneficial to *C. thermocellum* ethanol production from cellulose. In the co-cultures of cellulolytic *C. thermocellum* and non-cellulolytic *Thermoanaerobacter* strains (X514 and 39E), strain X514 improved ethanolic fermentation more effectively than strain 39E in co-culture, resulting in higher ethanol production by at least 62% in X514 co-cultures than in 39E co-cultures. Comparative genome sequence analysis revealed that the higher ethanol production efficiency of X514 compared with strain 39E was related to the presence of a complete vitamin B_12_ biosynthesis pathway (Figure 1C). Moreover, the ethanol production in strain 39E co-culture improved by 203% following the addition of exogenous vitamin B_12_ (Qiang et al., 2011).

### Interspecies Hydrogen and Electron Transfer

Interspecies hydrogen transfer that does not require physical interaction in cellulose-to-methane co-cultures combining cellulolytic Clostridia and methanogens has also been reported (Collet et al., 2003, 2005). In the co-culture of cellulolytic Clostridia and *Methanosarcina barkeri Fusaro*, *Methanosarcina mazei*, and *Methanothermobacter thermautotrophicus*, the methanogen utilized hydrogen and carbon dioxide, acetic acid, and even formic acid that was generated by the cellulolytic Clostridia from cellulose to produce methane (Sasaki et al., 2012; Lu et al., 2017). Moreover, interspecies hydrogen transfer between many hydrogen-producing *Clostridium* bacteria and methanogenic archaea has also been confirmed in the strong flavor baijiu ecosystem (Zou et al., 2018b). In addition, the interspecies hydrogen transfer interaction between acetogenic Clostridia (*C. aceticum*, *C. carboxidivorans*) and cellulolytic Clostridia/solventogenic Clostridia has also been confirmed. Acetogenic Clostridia convert H_2_ and CO_2_ that were produced by cellulolytic Clostridia/solventogenic Clostridia to acetate and butyric acid and butanol as the final products using the WLP (Xia et al., 2017; Youn, 2017).

A direct interspecies electron transfer mechanism has recently been characterized in *Geobacter* species, which maintain electron balance with other species through physical contact (Moscoviz et al., 2017). In co-culture systems of *C. pasteurianum* and *Geobacter sulfurreducens*, *C. pasteurianum* is the sole electron acceptor. *G. sulfurreducens* transfers electrons to *C. pasteurianum* and triggers a prominent metabolic shift of *C. pasteurianum*, which enhances 1,3-propanediol production at the expense of butanol and ethanol (Moscoviz et al., 2017). In addition, indirect interspecies electron transfer in co-culture of *C. beijerinckii* and *Geobacter metallireducens* has been reported in which *G. metallireducens* utilizes AQDS as the electron acceptor, regenerating extracellular electron shuttles of anthrahydroquinone-2, 6-disulfonate (AH_2_QDS), which is oxidized to AQDS by *C. beijerinckii*. AH_2_QDS regeneration resulted in changes in the intracellular NADH/NAD^+^ ratio in *C. beijerinckii* that enhanced biohydrogen production from xylose (Figure 1D) (Zhang et al., 2012).

### Direct Cell-to-Cell Material Exchange

Benomar et al. (2015) demonstrated that nutrient deficiencies of lactate and sulfate resulting in starvation of *Desulfovibrio vulgaris* triggered tight cell-to-cell interactions with Clostridia and material transfer. Physical evidence of aggregation between the two strains was observed by flow cytometry and was further investigated by fluorescence microscopy by labeling bacteria in the co-culture. In addition, a partial of peptidoglycan labelled with fluorochromes (green fluorescence) where the two strains occurred physical contact was observed by fluorescence microscopy and the tight cell-to-cell interactions were also observed by the SEM. When calcein-labeled *D. vulgaris* and *C. acetobutylicum* ATCC824 were co-cultured, calcein could be transferred in either direction between *D. vulgaris* and *C. acetobutylicum* cytoplasm. And when *D. vulgaris* with a pRD3 plasmid containing the gene mCherry (green fluorescent protein) was co-cultured with *C. acetobutylicum* without the mCherry gene but labeled with calcein, both bacteria exhibited both green and red fluorescence, which demonstrated they could exchange larger molecules such as proteins in the co-culture. In addition, the interspecies molecular exchange induced modifications of gene expression and changes in metabolism that resulted in an increase in hydrogen yield (Benomar et al., 2015). At the same time, Charubin et al. also confirmed direct cell-to-cell interactions and material exchange of acetone and acetoin between *C. acetobutylicum* ATCC824 and *C. ljungdahlii* ATCC55383 and, combined with direct electron transfer, it changed the gene-expression patterns of both partner organisms. This resulted in the production of non-native metabolites such as isopropanol and 2,3-butanediol (Figure 1E) (Charubin and Papoutsakis, 2019).

## Strategies to Enhance the Stability and Efficiency of *Clostridium* Co-Culture Systems

### Strategy for Optimizing the Fermentation Process

In the *Clostridium* co-culture system, the optimization of fermentation parameters such as pH, temperature, inoculum ratio, medium components, inoculation time, and control of anaerobic conditions plays an important role in the improvement and stability of co-culture yield. Among various parameters, the pH of the medium is one of the essential factors affecting cell growth and product yield; furthermore, changing the medium pH may induce a metabolic shift (Rabemanolontsoa et al., 2016). For example, it was demonstrated that high pH limits dark fermentation, and low pH limits light fermentation. Eventually pH 7 was determined to be the best condition for bacterial cooperation during a combined process to produce hydrogen through optimization of a co-culture of solventogenic Clostridia and anoxygenic phototrophic bacteria (Zagrodnik and Laniecki, 2015). In addition, adjustment of the phosphate buffer concentration (Laurinavichene and Tsygankov, 2016), adopting a fixed pH value (Zagrodnik and Laniecki, 2015), and addition of exogenous regulators such as eggshell biowaste (Pachapur et al., 2016a) were also shown to be good pH control strategies in many *Clostridium* co-culture systems. Moreover, co-culture of immobilized cells will be less affected by changes in medium pH and thus promote system stability and yield, which is also considered a good strategy (Xu and Tschirner, 2014).

Temperature control also has a great effect on the growth and production of the two bacteria in a co-culture and is an important optimization parameter in the fermentation process. Generally speaking, the common range of optimal temperature between the two bacteria is the parameter value range, and the optimal temperature value is obtained according to the fermentation performance. Another temperature control strategy is to inoculate high-temperature bacteria at high temperature for fermentation after a period of time, then lower the temperature to the temperature appropriate for the second bacterium, and then inoculate the second bacteria at that temperature for fermentation, such as in the co-culture of thermophilic *C. thermocellum* and mesophilic bacteria (Yu et al., 1985; Nakayama et al., 2011; Lin et al., 2013; Kiyoshi et al., 2015).

At the same time, the inoculation ratio is also considered when optimizing the product yield and improving the system stability in the *Clostridium* co-culture system. In the co-culture of *C. cellulovorans* and *C. acetobutylicum* from lignocellulosic substrates, under the condition of optimized inoculation rate at the inoculation ratio 5:3 (Cc: Ca), both strains established a synergistic relationship that resulted in the 2- to 3-fold hydrogen production improvement than the mono-cultures of *C. acetobutylicum* (Valdez-Vazquez et al., 2019). Meanwhile, inoculation time is usually optimized for a co-culture with cellulolytic Clostridia, especially in the co-culture of thermophilic cellulolytic *C. thermocellum* and mesothermal bacteria (Yu et al., 1985; Nakayama et al., 2011; Wen et al., 2014a, b), co-culture with facultative anaerobic bacteria (Yu et al., 1985; Nakayama et al., 2011; Wen et al., 2014a, b; Ebrahimi et al., 2019; Wu et al., 2019), and co-culture of solventogenic Clostridia and acetogenic Clostridia from CO_2_ and H_2_ (Youn, 2017). Li and Liu optimized the *C. thermosaccharolyticum* inoculation time in 125-mL anaerobic bottles and obtained the maximum hydrogen yield of 68.2 mL/g corn stalk when *C. thermosaccharolyticum* was inoculated 24 h earlier than *C. thermocellum*. And they successfully scaled up the hydrogen fermentation process from 125-mL anaerobic bottles to an 8-L continuous stirred tank reactor (Li and Liu, 2012).

Pachapur et al. (2017) demonstrated that each of the medium components played an important role in producing higher hydrogen in a co-culture system compared with a mono-culture. A complete metabolic pathway shift from a reductive to an oxidative pathway in glycerol fermentation was controlled by the medium composition, such as the concentrations of components KH_2_PO_4_ and MgSO_4_.7H_2_O, with decreased 1,3-propandiol (1,3-PD) production further increasing the hydrogen yield (Pachapur et al., 2017). Media components can act as activators to increase enzyme activity and also as cofactors for different enzymes during hydrogen production (Alshiyab et al., 2008). In order to meet the nutritional requirements and improve the yield of bacteria in co-culture, the composition of the medium was optimized, especially the substrate concentration (Ebrahimi et al., 2019). Kao et al. (2016a) utilized different sucrose concentrations of 4.45, 8.9, 17.8, and 35.6 g/L to investigate the effect on hydrogen production in the immobilized co-culture of *C. butyricum* and *R. palustris*. They found that the hydrogen production decreased gradually with sucrose concentration increasing from 4.45 g/L to 35.6 g/L, and the maximum hydrogen production of 5.42 mol/mol sucrose was acquired at 4.45 g/L sucrose, which showed high sucrose concentrations inhibited hydrogen production (Kao et al., 2016b). At the same time, the concentration of yeast extract has also been shown to have a significant effect on hydrogen production in co-culture of *C. butyricum* and *R. sphaeroides* (Laurinavichene and Tsygankov, 2016; Laurinavichene et al., 2016). In most cases, the co-culture medium contained growth factors necessary for the normal growth and metabolism of the two bacteria, or metal ions, trace elements and vitamins etc., in addition to different substrates, to maintain the stability of the co-culture and the performance of the fermented products (Ding et al., 2009). Recently, the method of mixing components of the medium at different proportions and selecting the best mixing ratio according to the fermentation performance was also used to optimize the medium, which is mainly explored in new co-cultures (Xia et al., 2017; Raut et al., 2019).

Due to differences in the metabolic capacities of *Clostridium* species in co-culture systems, the addition of different metabolites or cofactors can be used to regulate the metabolism of one or more microorganisms and further significantly influence the fermentation performance of the co-culture system. In a co-culture system of *C. thermocellum* and a non-cellulolytic *Thermoanaerobacter* strain (39E), the addition of 30 μg/L vitamin B_12_ improved ethanol production by 203% (Qiang et al., 2011). In addition, electron shuttling compounds, which are organic molecules that can cycle between oxidized and reduced forms and transfer electrons from a lower redox potential electron donor to a higher redox potential electron acceptor (Ye et al., 2011), may be added. Intracellular electron shuttles, such as nicotinamide adenine dinucleotide (NAD^+^), ubiquinone, ferredoxin, and cytochromes, play an essential role in the electron transport chain of microorganisms (Zhang, 2013). They were proved to increase the hydrogen yield and xylose utilization by increasing the acetate/butyrate ratio during acidogenic stages of *C. beijerinckii*. *C. beijerinckii* utilized electron shuttles to redirect carbon and electron flow to targeted pathways in the fermentative metabolism, which is a potentially viable strategy to improve hydrogen production in the co-culture system of *C. beijerinckii* and *G. metallireducens* (Ye et al., 2011; Zhang et al., 2012; Zhang, 2013). Alternative extracellular electron shuttles, including lawsone, juglone, indigo dye, humic acids, and fulvic acids, were also found to be able to substitute for AH_2_QDS, enhancing hydrogen production of the co-culture system (Zhang et al., 2013).

In the absence of an expensive reductant and nitrogen spraying, the synergies of co-culture systems can provide better process-based economic strategies for product production and larger field-scale applications. In addition, the elimination of a nitrogen sparging step was proved to be a process improvement strategy that resulted in changes in the metabolic pathways of the strain with increases of H_2_/ethanol/lactate and decreases of 1,3-PD production (Pachapur et al., 2015a). In a general way, facultative anaerobic bacteria will remove dissolved oxygen from the medium and oxygen from the reactor to provide an anaerobic environment for strictly anaerobic Clostridia in a closed reactor without reductants and nitrogen spraying. Zuroff et al. (2013) established a more stable obligate mutualism co-culture including *C. phytofermentans* and *S. cerevisiae* cdt-1 consuming oxygen to provide respiratory protection to the obligate anaerobe by controlling the volumetric transport rate of oxygen at approximately 8 μmol O_2_/L hour. Recently, a better companion bacterium was found that could not only remove oxygen but also exhibited an excellent synergistic effect, making it possible for Clostridia to ferment whether in an anaerobic environment or an aerated environment. The combination of *Nesterenkonia* and *C. acetobutylicum* formed a powerful “co-culture” for butanol production without oxygen removal measures before fermentation (Ebrahimi et al., 2019).

The pretreatment of substrates and the co-utilization of various substrates have also been shown to significantly improve the yield (Li et al., 2014; Pachapur et al., 2015b, 2016b; Rabemanolontsoa et al., 2016; Zhang et al., 2016b). Rabemanolontsoa et al. (2016) achieved a high carbon conversion efficiency of 84.9% utilizing hot-compressed water treatment of Japanese cedar in co-culture of *C. thermoaceticum* and *C. thermocellum* that produces acetic acid. The addition of Tween 80 to crude glycerol decreased the crude glycerol viscosity and improved its solubility and bioavailability, which improved the glycerol utilization rate and resulted in increasing hydrogen production by about 1.25-fold in the co-culture of *Enterobacter aerogenes* and *C. butyricum* from biodiesel waste (Pachapur et al., 2016b). Moreover, the co-culture of *Enterobacter aerogenes* and *C. butyricum* could also use crude glycerol to obtain hydrogen production of 26.07 ± 1.57 mmol H_2_/L of medium in the presence of apple pomace hydrolyzate as the indirect H-acceptor. A shift of metabolite pathway from the reductive to the oxidative route caused a production decrease of 1,3-PD and production increase of butyrate conducive to hydrogen production (Pachapur et al., 2015b). And Srivastava et al. (2018) utilized graphene oxide to treat thermostable crude cellulose that had been obtained via fungal co-cultivation of the strains *Cladosporium cladosporioides* NS2 and *Emericella variecolor* NS3 using a mixed substrate of orange peel and rice straw under solid state fermentation. Then they used sugarcane bagasse hydrolyzate from the graphene oxide-treated thermostable crude cellulose to co-culture *C. pasteurianum* MTCC116 and *Bacillus subtilis* PF_1 to obtain hydrogen production, which provided a novel strategy to enhance biohydrogen production (Srivastava et al., 2018).

### Cell Immobilization and New Fermentation Equipment

In addition to the optimization of the fermentation conditions, cell immobilization is also considered a potential strategy for improving the stability of the *Clostridium* co-culture system as it can provide moderate conditions for microbial growth and minimize the effects of system inhibitors (Laxman Pachapur et al., 2015). Jalil et al. (2018) gained a biohydrogen production rate of 35.9 mmol/h/L substrate utilizing pineapple biomass residue by using an immobilized co-culture of *C. sporogenes* ATCC19404 and *Enterobacter aerogenes* ATCC13048. They found that using an activated carbon sponge provided a better support material compared to loofah sponge, as they could enhance the production rate by approximately 67% compared to the free cell co-culture (Jalil et al., 2018).

The fermentation process and equipment have also been proved to be of great significance in improving the performance of *Clostridium* co-cultures (Table 5). Morsy developed a continuous fermentation system by controlling the hydraulic retention time to avoid substrate inhibition and achieved sustainable hydrogen production of 5.56 mol H_2_/mol hexose with *C. acetobutylicum* ATCC824 and *Rhodobacter capsulatus* DSM1710 (Morsy, 2017). And Li and Henson established a metabolic modeling of co-culture systems in anaerobic continuous stirred tank bioreactors (CSTBRs) to assess the CO-to-butyrate conversion in the co-culture of *C. autoethanogenum* and *C. kluyveri*. They predicted the system could enhance both butyrate productivity and titer (Li and Henson, 2019). Furthermore, good reactor and process design can not only achieve continuous and high yield product output, but also play a crucial role in maintaining the system stability through accurate monitoring and control of the culture in real time (Seppälä et al., 2011; Diender et al., 2019). The establishment of a continuously fed bioprocessing step involving in-line product extraction via gas stripping and product condensing within the syngas recirculation line achieved continuous butanol, hexanol, and octanol production from syngas and showed good stability in the co-culture of *C. ljungdahlii* and *C. kluyveri* (Richter et al., 2016).

**TABLE 5:** Strategies for enhanced *Clostridium* co-culture systems.

Regulation strategies	Products	Composition of *Clostridium* co-culture system	Results	References
Vitamin B_12_ addition	Ethanol	*C. thermocellum*, *Thermoanaerobacte*r strains 39E	Ethanol production was improved by 203% when adding 30 μg/L vitamin B_12_.	Qiang et al., 2011
Deletion of *Ldh* and *Pta* and adaptive evolution for 2,000 h	Ethanol	*C. thermocellum*, *Thermoanaerobacterium saccharolyticum*	A stable strain with 40:1 ethanol selectivity was obtained and the ethanol yield increased by 4.2-fold.	Argyros et al., 2011
Control of oxygen delivery (OTR)	Ethanol	*C. phytofermentans*, *S. cerevisiae* cdt-1	Maintenance of populations of 10^5^ to 10^6^ CFU/mL for 50 days.	Zuroff et al., 2013
Immobilization	Ethanol	*C. thermocellum* ATCC27405, *C. thermolacticum* ATCC43739	The ethanol yield increased by over 60% than free cell fermentation.	Xu and Tschirner, 2014
Optimization of substrate concentration, initial pH, and inoculum ratio	Hydrogen	*C. acidisoli* DSM12555, *R. sphaeroides* ZX-5	The yield of hydrogen was 10.16 mol/mol sucrose (5.08 mol/mol hexose).	Sun et al., 2010
Utilization of automatic experimental setting	Hydrogen	*C. butyricum*, *E. coli* K-12 MG1655	Improvement of experimental monitoring	Seppälä et al., 2011
Optimization of pH and utilization of a repeated fed-batch run	Hydrogen	*C. butyricum*, *R. sphaeroides*	The hydrogen production rate was 15.9 ml/L/h.	Lee et al., 2012
Utilization of continuous stirred tank reactor (CSTR)	Hydrogen	*C. thermocellum* DSM7072, *C. thermosaccharolyticum* DSM869	The yield of hydrogen was improved by 9.8%.	Li and Liu, 2012
Optimization of pH and utilization of 20 L batch bioreactors	Hydrogen	*C. butyricum*, *C. pasteurianum*	The yield of hydrogen was 2.91 mol/mol hexose.	Masset et al., 2012
Immobilization and optimization of initial pH	Hydrogen	*C. butyricum* ATCC19398, *R. palustris* ATCC17001	The yield was improved by 19.8%.	Kao et al., 2014
Dynamic microwave-assisted alkali pretreatment (DMAP) of cornstalk and optimization of the key factors affecting pretreatment process	Hydrogen	*C. thermocellum* DSM7072, *C. thermosaccharolyticum* DSM869	The effective removal of lignin and the released soluble compounds increased.	Li et al., 2014
Utilization of an up-flow anaerobic packed-bed reactor (APBR)	Hydrogen	*C. acetobutylicum* ATCC824, *Desulfovibrio vulgaris*	The hydrogen yield was 1.20 ± 0.26 mol/mol glucose.	Barca et al., 2016
Substrate pretreatment of adding Tween 80	Hydrogen	*C. butyricum* NRRL B-41122, *E. aerogenes* NRRL B-407	Hydrogen production increased around 1.25-fold in the presence of Tween 80.	Pachapur et al., 2016b
Substrate pretreatment of steam-exploded corn stover	Hydrogen	*C. cellulolyticum* DSM5812, *Citrobacter amalonaticus* Y19	The yield of hydrogen was 51.9 L/kg total solid.	Zhang et al., 2016b
Optimization of microorganism ratio and substrate and buffer concentrations	Hydrogen	*C. butyricum* NRRL-B1024, *R. palustris* GCA009	The yield of hydrogen was 6.4 ± 1.3 mol/mol glucose.	Hitit et al., 2017a
Utilization of continuous fermentation system (CFS)	Hydrogen	*C. acetobutylicum* ATCC824, *Rhodobacter capsulatus* DSM1710	The H_2_ yield was 5.65 mol/mol hexose.	Morsy, 2017
Immobilization	Hydrogen	*C. sporogenes* ATCC19404, *E. aerogenes* ATCC13048	The hydrogen production rate was 35.9 mmol/h/L_substrate_.	Jalil et al., 2018
Medium optimization	ABE	*C. butylicum* TISTR1032, *B. subtilis* WD161	The ABE yield was 9.71 g/L.	Tran et al., 2010
Medium optimization	ABE	*C. butylicum* TISTR1032, *B. subtilis* WD161	The ABE production was improved by 2.2-fold.	Tran et al., 2011
Optimization of inoculation timing, inoculation ratio, and pH control	ABE	*C. cellulovorans* 743B, *C. beijerinckii* NCIMB8052	The ABE yield were 19.9 g/L (acetone 3.96, butanol 10.9, and ethanol 5.04 g/L).	Wen et al., 2014a
Pretreatment feedstock of optimizing particle size	ABE	*C. thermocellum* ATCC27405, *C. beijerinckii* ATCC51743	Fermentation performance improvement of 670 mL gas.	Flythe et al., 2015
Acetate addition	ABE	*C. acetobutylicum*, *S. cerevisiae*	The Acetone concentration increased to 8.27–8.55 g/L, and the butanol concentration also increased to 13.91–14.23 g/L simultaneously.	Flythe et al., 2015
Exogenous cellulase enzyme addition	Butanol	*C. thermocellum* ATCC27405, *C. saccharoperbutylacetonicm* ATCC13564	The yiled of butanol was significantly increased to 6.9 g/L using 40 g/L of delignified rice straw.	Kiyoshi et al., 2015
Butyrate addition	Butanol	*C. acetobutylicum* ATCC824, *Saccharomyces cerevisiae*	The butanol concentration and butanol/acetone ratio were 15.74 g/L and 2.83, respectively.	Kiyoshi et al., 2015
Controlled oxygen delivery	Butanol	*C. acetobutylicum* TSH1, *B. cereus* TSH2	The yield of butanol was 11.2 g/L.	Wu et al., 2016
Utilization of an immobilized-cell fermentation system	Isopropanol and *n*-butanol	*C. beijerinckii* ATCC6014, *C. tyrobutyricum* ATCC25755	The yields of isopropanol and butanol were 6.78 and 12.33 g/L, respectively.	Zhang et al., 2016a
Continuous co-culture	Butanol, hexanol, and octanol	*C. ljungdahlii* PETC, *C. kluyveri* DSM555	The net rates of *N*-butyrate and *n*-caproate were 129 and 70 mmol C/L/d, respectively and the net rates of *n*-butanol, *n*-hexanol, and *n*-octanol were 39.2, 31.7, and 0.045 mmolC/L/d, respectively.	Richter et al., 2016
Addition of butyrate fermentative supernatant of *C. tyrobutyricum*	*n*-butanol	*C. acetobutylicum* ATCC824, *S. cerevisiae*	Final butanol and total ABE concentrations reached of 16.3 and 24.8 g/L.	Luo et al., 2017
Deletion of the cell division-related gene *maf* and neutral red addition, and temperature optimization	Butanol	*C. acetobutylicum* TSH1, *B. cereus* TSH2	The yield was 13.9 ± 1.0 g/L.	Mi et al., 2018
Optimization of inoculation amount/time and media formulation	Butanol	*C. acetobutylicum* PTCC1492, *Nesterenkonia sp*. F	The yield was 13.6 g/L.	Ebrahimi et al., 2019
Optimization of initial pH and inoculation amount/time of *S. cerevisiae*	Butanol	*C. beijerinckii*F-6, *S. cerevisiae*	The yield was 12.75 g/L and the productivity was 0.454 g/L/h.	Wu et al., 2019
Hot-compressed water treatment of Japanese cedar	Acetic acid	*C. thermoaceticum*, *C. thermocellum* ATCC27405	Conversion efficiency of 84.9%	Rabemanolontsoa et al., 2016
Utilization of two submerged hollow-fiber membrane bioreactors (s-HF/MBRs)	Caproic acid	*C. tyrobutyricum*, *Megasphaera hexanoica*	The yield was 10.08 g/L and the productivity was 0.69 g/L/h.	Kim et al., 2018
Utilization of continuous stirred-tank reactors (CSTR)	Chain elongated products	*C. autoethanogenum* DSM10061, *C. kluyveri* DSM555	The yields of butyrate and caproate were 5.5 ± 0.7 mM and 1.3 ± 0.3 mM, respectively.	Diender et al., 2019
Utilization of Continuous stirred tank bioreactor (CSTBR)	Butyrate	*C. autoethanogenum*, *Eubacterium rectale* or *C. kluyveri*	Higher volumetric productivities and lower substrate inhibition.	Li and Henson, 2019

### Genetic Modification

Various metabolic engineering tools have been developed for cellulolytic, solventogenic, acetogenic, and acidogenic Clostridia for gene overexpression, downregulation, and inactivation (Cheng et al., 2019). For example, the exogenous genes of *celA* and *celD*, which encode two glycoside hydrolases of *Neocallimastix patriciarum*, were separately cloned into *C. beijerinckii* NCIMB8052 (López-Contreras et al., 2001), which was applied in a *Clostridium* co-culture system. Antisense RNA for gene downregulation of the expression of the hydrogen-uptake hydrogenase gene (*hupCBA*) in *C. saccharoperbutylacetonicum* caused the *hupCBA* mutant strain to exhibit higher hydrogen production and lower butanol production compared to the parent strain *C. saccharoperbutylacetonicum* when co-cultured with *C. thermocellum*. The molecular selection of hydrogenase gene activity proved to be a potential strategy for strains producing higher butanol ratios (Nakayama et al., 2013). A NifA mutant strain resulting in constitutive nitrogenase activity and H_2_ production in *Rhodopseudomonas palustris* CGA676 was utilized to improve hydrogen production from cellulose in the *Clostridium* co-culture system (Jiao et al., 2012). Argyros et al. (2011) deleted the genes responsible for organic acid formation encoding lactate dehydrogenase (Ldh) and phosphotransacetylase (Pta) of *C. thermocellum* and obtained a stable strain with 40:1 ethanol selectivity and a 4.2-fold increase of ethanol yield over the wild-type strain after it evolved for 2,000 h. The co-culture of organic acid-deficient engineered strains of both *C. thermocellum* and *T. saccharolyticum* produced 38 g/L ethanol from 92 g/L avicel, with the production of acetic and lactic acids below detection limits, in 146 h (Argyros et al., 2011). At the same time, Mi et al. disrupted the *maf* gene with unknown function of *C. acetobutylicum* TSH1, and added *Bacillus cereus* TSH2 to form a new symbiotic co-culture system, resulting in an 8.9% butanol titer improvement from 12.3 ± 0.9 to 13.4 ± 1.1 g/L compared to the original symbiotic partnership (Mi et al., 2018). In addition, other different genetic manipulation strategies such as plasmid-based gene overexpression, antisense RNA for gene downregulation, gene inactivation via homologous recombination etc. are expected to be used to improve the stability and performance of co-culture systems through a better understanding of co-culture interactions at the molecular level based on systems biology methods.

## Conclusion

*Clostridium* co-culture systems can use extensive biomass-derived carbohydrates, fixing CO_2_ autotrophically, enabling maximum carbon substrate utilization, and achieving very high metabolite yields (Charubin et al., 2018), making them of great significance to the production of many important products, such as hydrogen, butanol, and methane, and energy development. Further improvements to the productivity of co-culture systems will require a comprehensive understanding of the physiological characteristics and interaction mechanisms of individual members and entire co-culture systems. Fortunately, genome sequencing technology has greatly improved our understanding of the functions and genetic background of *Clostridium* species (Zou et al., 2018b). Other systems biology approaches could also be applicable to the comprehensive analysis of the physiological characteristics of Clostridia in co-culture systems (Ivey et al., 2013; Lu et al., 2017). Furthermore, genome-scale metabolic models, which contain all the metabolic information of a single organism or ecosystem (Zhang et al., 2013; Moscoviz et al., 2017), can be used to understand physiological characteristics and interspecies interactions (Tran et al., 2010). Salami et al. (2010) developed a genome-scale metabolic model of *C. cellulolyticum* in a co-culture with *C. acetobutylicum*, and suggested that cellobiose inhibition is not the main factor responsible for improved cellulose utilization compared with the mono-culture of *C. cellulolyticum*. Systems biology could contribute to major advances in the understanding of fermentation regulation, molecular modifications, and synthesis of artificial micro-ecosystems in the future. Combining physiological experiments, molecular analysis, systems biology, and bioinformatics will allow us to fully understand the interaction mechanisms of co-culture systems, and the use of synthetic biology to build new artificial microbial ecosystems for the production of desired products is likely to be a trend in the future.

## Author Contributions

YD and WZ conceived, designed, and wrote the manuscript. WZ, KZ, GY, and JY helped to draft and revise the manuscript. All the authors read and approved the final manuscript.

## Conflict of Interest

The authors declare that the research was conducted in the absence of any commercial or financial relationships that could be construed as a potential conflict of interest.

## Abbreviations

DFE: dark fermentation effluent
CBP: consolidated bioprocessing
OTR: oxygen transfer rate
CFU: colony forming unit
ABE: acetone-butanol-ethanol
FISH: the method of fluorescence in situ hybridization
QPCR: quantitative polymerase chain reaction
RT-qPCR: quantitative reverse-transcription PCR
SEM: the scanning electron microscopy
WLP: the wood-ljungdahl pathway
MCC: microcrystalline cellulose
AQDS: anthraquinone-2, 6-disulfonate
AH_2_QDS: anthrahydroquinone-2, 6-disulfonate
ATP: adenosine triphosphate
ADP: adenosine diphosphate
AMP: adenosine monophosphate
NADH: nicotinamide adenine nucleotide
NAD^+^: nicotinamide adenine dinucleotide
COD: chemical oxygen demand
1,3-PD: 1,3-propanediol
CH_4_: methane
CO: carbon monooxide
CO_2_: carbon-dioxide
H_2_: hydrogen
TS: total solid
CSTBR: continuous stirred tank bioreactor
*Ldh*: lactate dehydrogenase
*Pta*: phosphotransacetylase
DMAP: dynamic microwave-assisted alkali pretreatment
APBR: an up-flow anaerobic packed-bed reactor
CFS: continuous fermentation system
S-HF/MBRs: two submerged hollow-fiber membrane bioreactors
CSTR: continuous stirred-tank reactors.
